# Cytokine responses to *Staphylococcus aureus*bloodstream infection differ between patient cohorts that have different clinical courses of infection

**DOI:** 10.1186/s12879-014-0580-6

**Published:** 2014-11-15

**Authors:** Sinead McNicholas, Alida Fe Talento, Joanne O’Gorman, Margaret M Hannan, Maureen Lynch, Catherine M Greene, Hilary Humphreys, Deirdre Fitzgerald-Hughes

**Affiliations:** Department of Clinical Microbiology, RCSI Education and Research Centre, Beaumont Hospital, Dublin 9, Ireland; Department of Microbiology, Beaumont Hospital, Dublin, Ireland; Department of Microbiology, Mater Misericordiae University Hospital, Dublin, Ireland; Department of Medicine, Royal College of Surgeons in Ireland, Dublin, Ireland

**Keywords:** Staphylococcus aureus, Bloodstream infection, Pro inflammatory cytokine, Renal impairment, Haemodialysis, Immune mediator, Leptin, RANTES, IL-6

## Abstract

**Background:**

The clinical course of *Staphylococcus aureus* bloodstream infection is unpredictable and bacterial virulence, host immune response and patient characteristics are among the factors that contribute to the clinical course of infection. To investigate the relationship between cytokine response and clinical outcome, circulating cytokine levels were investigated in response to *S. aureus* bloodstream infection in patients with different clinical courses of infection.

**Methods:**

A prospective study was carried out in 61 patients with *S. aureus* bloodstream infection and circulating levels of IL-6, GRO-γ, RANTES and leptin were assessed over the course of the infection. Levels were compared in patients with complicated courses of infection (e.g. infective endocarditis) *versus* uncomplicated courses of *S. aureus* bloodstream infection and methicillin-resistant *S. aureus Vs* methicillin-susceptible *S. aureus* infection.

**Results:**

Significantly lower leptin levels (*p* < 0.05) and significantly higher IL-6 levels (*p* < 0.05) were detected at laboratory diagnosis in patients with complicated compared to uncomplicated *S. aureus* bloodstream infection. Significantly higher levels of GRO-γ were associated with MRSA infection compared to MSSA infection.

**Conclusions:**

IL-6 may be an early inflammatory marker of complicated *S. aureus* bloodstream infection. Leptin may be protective against the development of a complicated *S. aureus* bloodstream infection.

**Electronic supplementary material:**

The online version of this article (doi:10.1186/s12879-014-0580-6) contains supplementary material, which is available to authorized users.

## Background

*Staphylococcus aureus* bloodstream infection (SABSI) is one of the most severe manifestations of *S. aureus* with an estimated mortality rate of 20% [1]. However, the outcome of SABSI is variable. Many patients develop uncomplicated resolving infections that respond to appropriate antimicrobial therapy while others develop persistent or complicated infection with metastatic foci (e.g. infective endocarditis (IE)), which may be fatal. Factors that contribute to the clinical outcome of SABSI, include the virulence and antibiotic susceptibility of the infecting isolate [2],[3], host innate and humoral immune responses [4],[5] and the underlying condition of the patient [6]. *S. aureus* molecules such as peptidoglycan and lipoteichoic acid (LTA) are potential stimulators of cytokine production (e.g. TNF-α, IL-1β, IL-6, IL-4, IL-8, IFN-γ and IL-12), in response to infection [7] but un-regulated cytokine production may contribute to *S. aureus* pathogenesis. Differential cytokine responses to *S. aureus* may contribute to varying clinical outcomes of SABSI. This prospective study aimed to; identify cytokines or chemokines that are important in the immune response to SABSI, determine the levels of these molecules in sequential plasma samples taken over the course of SABSI and investigate these cytokine responses in SABSI with a complicated clinical course *versus* an uncomplicated course and those with MRSA infection compared to those with MSSA infection.

## Methods

### Setting, patients and definitions

Blood samples were collected from patients with SABSI in Beaumont Hospital (BH), from October 2008 to February 2011 and in the Mater Misericordiae University Hospital (MMUH) from November 2009 to March 2010. Both are >500-bed tertiary referral centres, located in Dublin, Ireland. BH is the national centre for renal and pancreatic transplantation, neurosurgery and cochlear implantation. The MMUH has the largest cardiothoracic surgical department in the country and is the national centre for heart and lung transplantation. Approval from BH and MMUH Ethics Committees was received. Participation was by informed consent/assent from patients/relatives. Patient demographics, including age, sex, co-morbidities, timing of onset of SABSI, source of SABSI, isolate antimicrobial susceptibility and infection outcomes were collected prospectively from patient's charts, nursing notes and microbiological records. Plasma samples were prepared from blood collected on the day of the laboratory diagnosis of SABSI, seven days later and in patients with complicated infection, on day 14 after laboratory-confirmed diagnosis. Plasma was also prepared from the blood of four healthy control individuals. An uncomplicated course of infection was defined specifically for this study as follows; negative follow-up blood culture, subsidence of fever within 72 h, normal findings on transesophageal echocardiogram and no symptoms suggestive of metastatic infection. A complicated course of infection was defined specifically for this study as; positive follow up blood culture, despite at least three days of appropriate antibiotics (e.g. flucloxacillin for methicillin-susceptible *S. aureus* (MSSA), vancomycin for methicillin-resistant *S. aureus* (MRSA)), disseminated infection such as osteomyelitis, or IE. Nosocomial, healthcare-associated and community-acquired infection were defined as described by Friedman *et al*. [8].

### Preliminary identification of cytokines in plasma from patients with SABSI using a panel of pro-inflammatory cytokines

A commercially available antibody based cytokine/chemokine microarray (RayBio® Human Cytokine Antibody Array 3 (RayBiotech Inc., GA, USA)) was used to identify cytokines that were differentially expressed in pooled plasma (taken on the day of diagnosis) from three patients with uncomplicated BSI and three with complicated BSI. Pooled plasma was used for the preliminary screen only, to minimise resources required. The panel consists of antibodies against 42 pro-inflammatory cytokines and chemokines and the manufacturer's instructions were followed. Cytokine signal intensities were captured and quantified by the chemiluminescence imaging system G:BOX Chemi XL (Syngene UK, Cambridge, UK). Mean values for each cytokine were expressed relative to the mean positive value for each array membrane (mean of 6 positive control spots per membrane). A change in cytokine levels in pooled plasma, at a ratio threshold of at least 1.4 fold between groups was considered to be potentially relevant and the results from this preliminary screen influenced the decision on which cytokines to investigate in individual patients over the course of their infection.

### Enzyme-linked immunosorbant assay (ELISA)

The levels of IL-6, GRO-γ, RANTES and leptin were determined in patient and healthy donor plasma samples using immunometric sandwich ELISA (R and D systems, Abington, UK (IL-6, RANTES, leptin) or Acris Antibodies Ltd, Germany (GRO-γ). The manufacturer's instructions were followed using 50–100 μl of plasma. Plasma protein concentrations were measured using the Bradford assay and cytokine levels were normalised to plasma protein concentration to account for variability in blood processing and biological variations in plasma protein concentrations between patients, many of whom were undergoing haemodialysis.

### Statistical analysis

Unpaired *t*-tests were used to compare the means of data sets. Fisher's exact test was used to compare categorical data. Graphpad Prism 4 was used for Statistical analyses.

## Results

### Patient details

In total, 61 patients were studied, 15 (24.5%) with MRSA BSI, 46 (75.4%) with MSSA BSI. Fifty (82%) patients had an uncomplicated BSI and 11 (18%) had a complicated course of SABSI as defined for this study. Detailed patient demographics are shown in Table 1. Two patients died and SABSI was identified as the cause in one of these (complicated, IE). A high rate of IE was found among those with complicated SABSI (8/11, 73%) and most complicated SABSI was healthcare-associated (7/11, 64%). Of the 15 patients with MRSA infection, four had a complicated clinical course and an increased risk of complications was not identified in those with MRSA *Vs* MSSA (4/15, 26% Vs, 11/46 (24%), RR1.1).

**Table 1:** **Characteristics of 61 patients with** ***S. aureus*** **bloodstream infection (BSI)**

Patient characteristic	No. of patient (%)
**Gender**	
Male	38 (62.3)
Female	23 (37.7)
**Age**	
>65	35 (57.38)
<65	26 (42.63)
**Co-morbidities** ^***a***^	
Cardiac disease	22 (36.07)
^*b*^Renal impairment	23 (37.70)
Diabetes mellitus	16 (26.23)
Chronic obstructive pulmonary disease	8 (13.11)
Autoimmune disease	6 (9.84)
**Onset of BSI** ^*c*^	
Nosocomial	31 (50.82)
Healthcare-associated	22 (36.07)
Community-acquired	8 (13.11)
**Source of BSI**	
Unknown	15 (24.6)
Prosthetic device	30 (49.18)
Other	16 (26.23)
**Susceptibility of infecting isolate**	
Methicillin-susceptible *S. aureus*	46 (75.41)
Methicillin-resistant *S. aureus*	15 (24.59)
**Outcome of BSI**	
Uncomplicated BSI	50 (81.96)
Complicated BSI	11 (18.03)
Death	2 (3.28)
**Type of complication**	
Infective endocarditis	8 (13.11)
Persistently positive blood culture	1 (1.64)
septic arthritis	1 (1.64)
Discitis	1 (1.64)

^*a*^More than one co-morbidity was indentified in some patients. ^*b*^Of 23 patients with renal impairment, 21 were on haemodialysis (HD), one had chronic renal failure but was not on HD and one was receiving plasmaphoresis for Waldenströms macroglobulinaemia. ^*c*^Onset of BSI defined as described by Friedman *et al*., 2002 [8].

### Identification of cytokines for further investigation in complicated or uncomplicated SABSI

**Figure 1:**
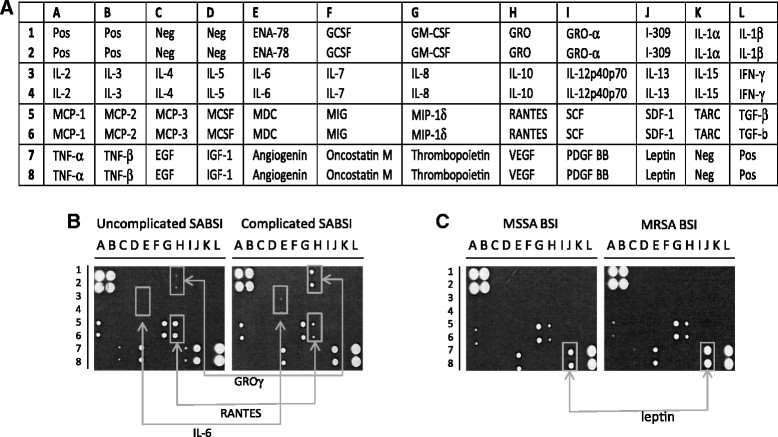
**Analysis of differential cytokine levels in pooled plasma from patients with SABSI.** The cytokine antibody array map (RayBiotech Inc., GA) showing the positions of 42 duplicate cytokines, positive and negative controls **(A)**. Hybridization patterns for pooled plasma from three patients *per* group, comparing patients with complicated SABSI to patients with uncomplicated SABSI **(B)** and patients with MSSA to patients with MRSA **(C)**. Arrows indicate cytokines selected for investigation in all patients (**B** and **C**) and were based on a fold change in levels of ≥1.4 between groups. Fold change was estimated from cytokine signal intensity from compared groups (uncomplicated/complicated, MSSA/MRSA) where signal intensity was normalised with respect to the signal intensity of six positive control spots on each membrane, located at positions A1, A2, B1, B2, L7, L8.

A panel of 42 cytokine antibodies were probed by hybridization of pooled plasma taken on the day of diagnosis, from three patients with complicated SABSI and three with uncomplicated SABSI. The position and name of each of the 42 cytokine antibodies on the array is shown on the cytokine antibody array map (Figure 1A). An image of the resulting hybridisation patterns for representative pooled samples from each group is shown in Figure 1B and cytokines that showed ≥1.4 fold differences in levels between groups are highlighted. These cytokines; IL-6, GRO-γ and RANTES (Regulated upon Activation, Normal T-cell Expressed, and Secreted) were identified for further study. Leptin levels were marginally lower in pooled plasma from patients with complicated SABSI compared to uncomplicated but this cytokine was further studied because its levels were increased >2-fold in pooled plasma from three patients with MRSA compared to MSSA BSI (Figure 1C).

**Figure 2:**
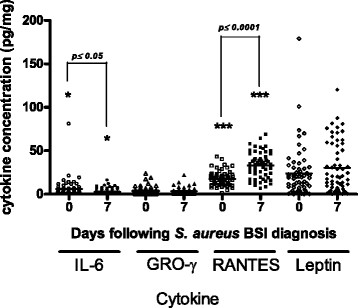
**Scatter plot of the concentrations of four cytokines in all patient samples collected over the course of** ***S. aureus*** **BSI.** Cytokine levels on the day of laboratory diagnosis and seven days after diagnosis of SABSI are shown (n = 61). Horizontal bars show the mean values for each cytokine.

Further investigation of these four cytokines in all patients with SABSI, using individual ELISAs revealed inter-patient variability in cytokine levels. However, the mean cytokine levels varied over the course of SABSI (Figure 2). Mean IL-6 and GRO-γ levels decreased over the course of infection, and the decrease was statistically significant in the case of IL-6 (*p* ≤ 0.05). Mean RANTES levels were increased seven days following laboratory diagnosis compared to levels on the day of laboratory diagnosis of SABSI (*p* ≤ 0.0001). Further samples were taken 14 days following SABSI diagnosis only from patients with complicated SABSI (n = 11) and were therefore not included in these analyses.

### Cytokine levels and the clinical outcome of SABSI

**Figure 3:**
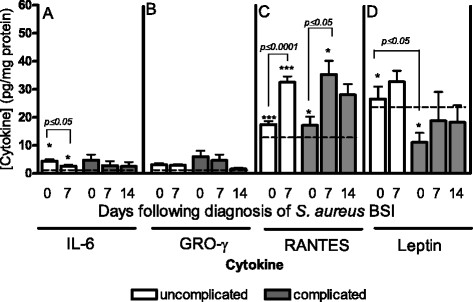
**Cytokine levels in patients with uncomplicated** ***Vs*** **complicated SABSI.** Cytokine levels in plasma from patients with uncomplicated (clear bars, n = 50) and complicated (solid grey bars, n = 11) SABSI at diagnosis (day 0), day 7 and day 14. Data shown represent mean cytokine concentration ± SEM for IL-6 **(A)**, GRO-γ **(B)**, RANTES **(C)** and leptin **(D)**. Dotted lines in each panel indicate the mean cytokine levels found in four healthy control individuals.

The mean cytokine levels found in patients with complicated SABSI Vs uncomplicated SABSI are shown in Figure 3. Those with complicated SABSI had significantly higher mean IL-6 levels compared to those with uncomplicated SABSI on the day of diagnosis (*p* ≤ 0.05) (Figure 3, panel A). Mean GRO-γ levels were greater in patients with complicated SABSI compared to uncomplicated SABSI but these differences were not statistically significant (Figure 3, panel B). Mean RANTES (Figure 3, panel C) levels were significantly higher on day seven following diagnosis of SABSI compared to those found on the day of diagnosis in patients with uncomplicated (*p* ≤ 0.0001) and complicated SABSI (*p* ≤ 0.05). Leptin levels (Figure 3, panel D) were significantly reduced in patients with complicated SABSI compared to patients with uncomplicated SABSI on the day of diagnosis (*p* ≤ 0.05). Mean levels of IL-6, GRO-γ and RANTES values in healthy individuals were lower than those in patients with complicated and uncomplicated SABSI on the day of diagnosis and seven days later. Mean leptin levels, although lower in healthy individuals than those with uncomplicated SABSI, were raised in healthy individuals compared to those with complicated infection (Figure 3).

### Cytokine responses SABSI caused by MSSA Vs MRSA

**Figure 4:**
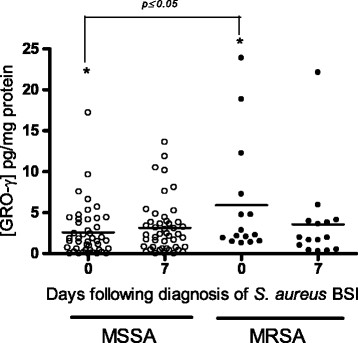
**Scatter plot of GRO-** **γ** **levels in patients with SABSI caused by MRSA** ***Vs*** **SABSI caused by MSSA.** GRO-γ levels in patients with MSSA (open circles, n = 46) or MRSA BSI (closed circles, n = 15) over the course of SABSI. Horizontal bars show the mean values for each cytokine.

The mean levels of GRO-γ were significantly greater in patient with SABSI in cases where an MRSA was the source of infection (p ≤ 0.05) but this increase was only evident when comparing the samples taken on the day of diagnosis (Figure 4). The mean level of the other three cytokines investigated, were not significantly altered in patients with SABSI caused by MRSA *Vs* MSSA (data not shown).

## Discussion

Pro-inflammatory cytokines released in response to *S. aureus* infection may contribute to the cascade of events that lead to severe complications in SABSI [9]. Cytokines including IL-6, GRO-γ and RANTES are up-regulated in response to *S. aureus* clinical isolates in epithelial cell infection models [10],[11]. We observed changes in the circulating levels of these cytokines in patients over the course of SABSI. In addition, greater circulating IL-6 levels and reduced leptin levels were observed in complicated SABSI compared to uncomplicated SABSI. Complicated infection was defined specifically for this study as described above and IE was the predominant complication. The surprisingly high rate of IE (8/61, 13%) may be partly explained by our policy of echocardiograms for all patients with SABSI regardless of the response to therapy and the presence of a national centre for cardiac and lung transplantation in one of the study hospitals. In addition, an increased circulating level of GRO-γ was noted in response to MRSA Vs MSSA bloodstream infection.

The cytokine response to sepsis has been well documented, with IL-6 suggested as an early marker and a marker of severity [12]-[15]. The cytokine response in SABSI also appears to involve IL-6 elevation early in the course of infection although the levels were lower than those reported in Gram-positive sepsis [16]. An earlier study suggested that IL-6 levels decay more rapidly in sepsis survivors than in non-survivors over a 96 h period supporting its prognostic value [17]. However, in the present study on SABSI, where cytokines were assessed over a longer period, IL-6 levels decreased to similar levels in complicated and uncomplicated SABSI by day seven following laboratory diagnosis. Nonetheless, a significantly elevated IL-6 level on the day of diagnosis of SABSI appears to correlate with a poorer prognosis.

*In-vitro* studies have shown increased RANTES levels in epithelial cells exposed to *S. aureus* clinical isolates [11]. However, a role for RANTES in the immune response to SABSI has not been previously reported. RANTES’ main function is as a chemotactic agent for leucocytes facilitating their recruitment to infection sites [18]. RANTES production is thought to be induced by TNF-α and IL-1α, both of which are produced in response to *S. aureus* components (e.g. peptidoglycan, LTA) [19],[20]. Our observation of a significant rise in RANTES levels in all patient groups by day seven, suggests that RANTES production is stimulated by *S. aureus in vivo* and it may have a functional role in the immune response to this pathogen. It appears that once produced, circulating RANTES levels remain high. Furthermore, mean levels remained elevated in those with complicated SABSI up to 14 days following diagnosis. The purpose of this persistence is not clear but it may reflect the ongoing response to persistent SABSI.

Leptin levels were significantly higher in uncomplicated *versus* complicated SABSI on the day of laboratory diagnosis (*p* ≤ 0.05) and a similar trend was identified on day seven following diagnosis (*p* = 0.071), indicating that patients with more severe infection had a sustained attenuated leptin response. Leptin is reported to have both beneficial and detrimental effects for the host in the setting of sepsis. It stimulates proliferation of lymphohaematopoetic cells and increases the phagocytic activity of macrophages which may enhance host eradication of *S. aureus* but it can also activate endothelial cells resulting in a severe sepsis phenotype [21],[22]. Both negative and positive correlations between leptin levels and clinical outcome in patients with sepsis have been reported [22],[23]. The present study supports the suggestion that higher leptin levels are protective. Decreased leptin levels in patients with complicated infection may contribute to poor clinical outcome by reducing the host's ability to eradicate the infection. These findings support the possibility that administration of leptin may benefit patients with severe SABSI as one animal study reported that exogenous administration of leptin reduced the severity of *S. aureus*-induced septic arthritis in two mouse models [24].

We did not find evidence that GRO-γ levels were altered in patients with a complicated course of infection. However, our data suggest that this cytokine may play a role in the response to MRSA infection specifically. The cause and clinical implications of this finding are unclear. Bacterial components, such as peptidoglycan and LTA that stimulate GRO-γ production via IL-1 and TNF-α [20] are common to MRSA and MSSA. Furthermore, the chemoattractant properties of GRO-γ are equally important in MRSA and MSSA (BSI). It has been shown that *mecA* augments the expression of virulence genes such as *fnb*[25] and increased expression of α-toxin and phenol soluble modulin has been shown in community-acquired MRSA [26]. It is therefore possible that differential host immune responses such as increased GRO-γ production may result indirectly from increased expression of virulence genes in MRSA.

There were limitations to this study. Despite extension to two centres, only 61 patients were recruited over 28 months, providing a relatively small study size. There were logistical issues related to obtaining samples from very ill patients or the movement or discharge of patients before samples could be taken. Samples at 14 days after diagnosis were only taken from inpatients with complicated SABSI and not those discharged by this time. Moreover, a true baseline cytokine level was not obtained as this would require screening large numbers of patients before the development and laboratory diagnosis of SABSI. Complicated SABSI was under-represented because of the difficulty obtaining patient consent or assent from relatives of very ill or rapidly deteriorating patients. The contribution of co-morbid conditions associated with SABSI (e.g. concurrent infection, autoimmune conditions, recent surgery etc.) which was not part of this study, to the cytokine response cannot be excluded. Finally, the first blood sample was taken on the day of laboratory diagnosis of SABSI and not when SABSI was first suspected on clinical grounds. This time difference was variable but may have been at least two days, over which cytokine levels may have changed.

## Conclusions

In conclusion, differential expression of certain cytokines, such as IL-6 and leptin occurs in patients with different clinical courses of SABSI. While acknowledging that further studies on larger patient groups are required, these cytokines may be considered as potential prognostic markers or potentially be modified to minimise the adverse effects of *S. aureus* infection in vulnerable patients. Additionally, the increased expression of RANTES in all groups over the course of SABSI suggests that it may play a novel role in response to this pathogen.

## Authors' contributions

SMN participated in the conduct of the experiments described and produced the first draft. AT and JO’G participated in patient recruitment and sample collection, MH and ML facilitated the study, guided the ethics application at MMUH and provided critical appraisal of the manuscript, CG participated in data analysis and interpretation of the data and provided critical appraisal of the manuscript. HH and DFH conceived of the study, DH directed the experimental work and redrafted the manuscript. All authors approved the final manuscript.

## Authors’ original submitted files for images

Below are the links to the authors’ original submitted files for images. Authors’ original file for figure 1 Authors’ original file for figure 2 Authors’ original file for figure 3 Authors’ original file for figure 4

## Abbreviations

SABSI: *Staphylococcus aureus* bloodstream infection
RANTES: Regulated upon activation, normal T-cell expressed
GRO: Growth regulated oncogene
IE: Infective endocarditis
MRSA: Methicillin-resistant *S. aureus*
MSSA: Methicillin-susceptible *S. aureus*
TNF: Tumour necrosis factor
IL: Interleukin
IFN: Interferon
LTA: Lipoteichoic acid
ELISA: Enzyme linked immunosorbant assay
BH: Beaumont Hospital
MMUH: Mater Misericordiae University Hospital
