# Supplementary figures and images for: Cytokine responses to Staphylococcus aureusbloodstream infection differ between patient cohorts that have different clinical courses of infection
Source: BMC Infect Dis. 2014 Nov 15;14:580. doi: 10.1186/s12879-014-0580-6 (PMC4237739; doi:10.1186/s12879-014-0580-6)

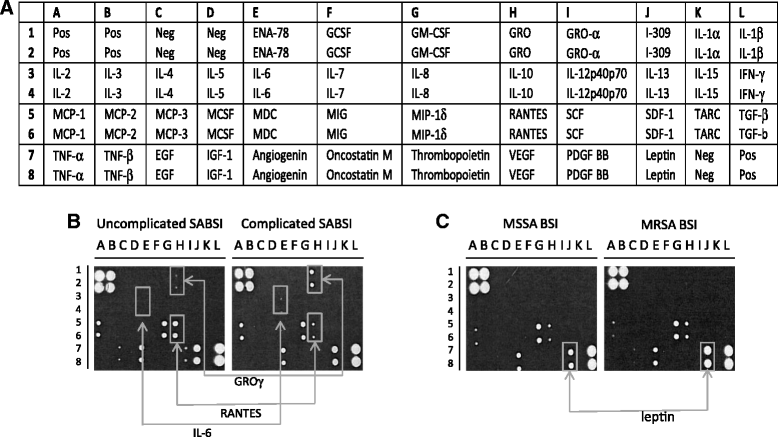

Supplement: Supplementary file 1 — Authors’ original file for figure 1 [file 12879_2014_580_MOESM1_ESM.gif]

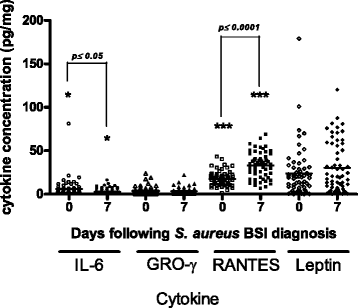

Supplement: Supplementary file 2 — Authors’ original file for figure 2 [file 12879_2014_580_MOESM2_ESM.gif]

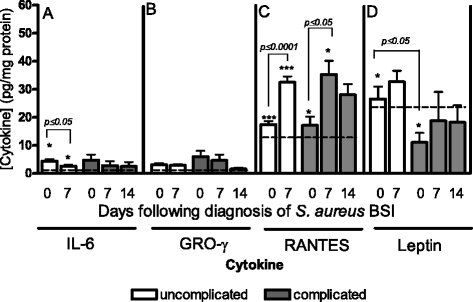

Supplement: Supplementary file 3 — Authors’ original file for figure 3 [file 12879_2014_580_MOESM3_ESM.gif]

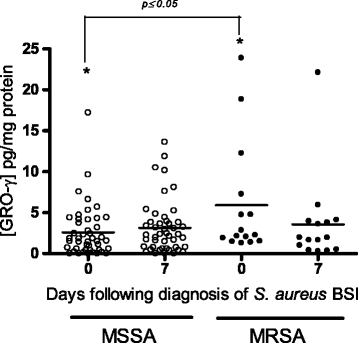

Supplement: Supplementary file 4 — Authors’ original file for figure 4 [file 12879_2014_580_MOESM4_ESM.gif]
